# PROTOCOL: The effectiveness, implementation and cost effectiveness of mentoring programmes in reducing anti‐social, violent and offending behaviour in children aged 17 years and below: A mixed method systematic review

**DOI:** 10.1002/cl2.1286

**Published:** 2022-11-12

**Authors:** Monisha Lakshminarayanan, Guy Skinner, Jing Li, Patrick Tolan, David Du Bois, Howard White

**Affiliations:** ^1^ Campbell South Asia Delhi India; ^2^ Campbell Collaboration Cambridge UK; ^3^ Evidence‐Based Social Sciences Research Center, School of Public Health Lanzhou University Lanzhou China; ^4^ Department of Psychiatry, Institute for Juvenile Research University of Illinois Chicago Illinois USA; ^5^ Institute for Health Research and Policy, School of Public Policy University of Illinois Chicago Illinois USA; ^6^ Global Development Network Delhi India

## Abstract

This is the protocol for a Campbell systematic review. The review will address the following research questions: What is the evidence on the effects of adult mentoring programmes in reducing anti‐social, violent and offending behaviour in children aged under 18 years? Are these effects sustained after the end of mentoring? Which aspects/features of adult mentoring programmes promote the reduction of anti‐social, violent and criminal behaviour in children aged under 18 years? What are the hindering factors/barriers that affect the successful implementation of adult mentoring programmes in children aged under 18 years? What are the supporting factors/facilitators that contribute to the successful implementation of adult mentoring programmes in children aged under 18 years? What is the evidence on programme costs and incremental cost effectiveness? (The incremental (or marginal cost) is the cost of providing the intervention over and above the cost of usual services).

## 1 BACKGROUND

Youth participation in offending, anti‐social and violent behaviour is a significant cause of concern. There has been a significant increase in serious violence, as well as the number of knife‐related crimes committed by children in recent years (White et al., 2021). Youth violence is the fourth leading cause of death for young people worldwide, with an estimated 200,000 deaths per year (World Health Organization, 2015). The problem affects victims and perpetrators of youth violence, their families, friends, and communities to a great extent.

Over the last decade, the number of research studies describing risk factors that contribute to youth violence, as well as protective factors that reduce victimisation and perpetration rates, have seen a gradual rise (World Health Organization, 2015).

Programmes may take either a deterrence or rehabilitation approach to address behavioural problems in juveniles as they negotiate the transition from childhood to adulthood (Lipsey et al., 2010). Previous reviews of existing evidence demonstrated that many such programmes have been effective in preventing or reducing offending (e.g., Lipsey & Cullen, 2007).

Mentoring interventions have been identified as one such effective intervention for high‐risk youth or youth engaged in anti‐social behaviours (DuBois et al., 2002; Raposa et al., 2019; Tolan et al., 2008). Mentoring interventions connect children at‐risk with older children or adults to facilitate the development of supportive, empathetic, and healthy relationships which are intended to lead to positive child outcomes and a range of desired behaviour changes. For the purposes of this review, offending is defined as relating to the committing of an illegal act or the break of a law. Antisocial behaviour is defined as behaviour by a person which causes, or is likely to cause, harassment, alarm, or distress to persons not of the same household as the person.

There is a good evidence base available for mentoring interventions in children and adolescents. The outcome measures in the existing reviews on mentoring relate to different domains of child development, education, and health (Christensen et al., 2020; Du Bois et al., 2011; Raposa et al., 2019). The reviews which consider offending outcomes requires updating (Tolan et al., 2013). The existing evidence reviews are either exclusively quantitative in nature or narrative syntheses, rather than the mixed methods approach which will be taken in this review. Our mixed methods review approach will thus assess both effectiveness and implementation evidence on adult mentoring for children who display or are at risk of displaying violent, anti‐social or offending behaviour. We will also include evidence from studies that report cost data in our review.

The proposed review will assess the effectiveness of mentoring programmes run by adults in reducing anti‐social, violent, and criminal behaviour in children aged under 18 years.

### 1.1 The intervention

Mentoring has been described by the United States Office of Juvenile Justice and Delinquency Prevention as an unwavering, altruistic relationship between an elder and more experienced peer and a novice or inexperienced youth. Recent times have seen an upward trend in mentoring activities (Garringer et al., 2017).

Whilst the nature of formal mentoring interventions and their components vary (Karcher & Hansen, 2014), four key common characteristics have been identified (Tolan et al., 2013): (1) The recipient's identification with the mentor, which aids in motivation, behaviour and bonding; (2) information or training to help with social, educational, legal, family, and peer difficulties; (3) advocacy for the mentee in many systems and settings; and (4) emotional support and friendliness to build self‐efficacy, confidence, and a sense of belonging (Tolan et al., 2013).

Mentoring can be split into two categories: formal and informal mentoring (Chao et al., 1992). In the case of formal mentoring a mentor is recruited, trained and matched with a mentee to engage in various activities and address risk behaviours. Informal mentoring also known as natural mentoring evolves organically from the youth's social environment and the mentoring process is largely unstructured in nature (Rhodes & DuBois, 2008). Examples of informal mentors could include teachers, sports coaches and religious figures.

Other distinctions are between adult or peer mentoring and between one‐one or group mentoring interventions (Burton, 2020) as well as in‐person versus virtual forms of mentoring, whether programmes take place at a specific site (e.g., school) or allow for mentor‐youth activities to take place in a variety of communities settings, whether additional components (e.g., skills training or stipends) are included distinct from and in addition to mentoring, and whether mentors are volunteers or paid (Garringer et al., 2017). Garringer et al. (2017) and Tolan et al. (2013) also highlight the importance of long mentor‐mentee relationships, the need for consistent and regular meetings, and whether the mentors receive training and supported throughout the mentoring process.

Lastly, there are non‐structured approaches versus structured approaches. The traditional approach is non‐structured or non‐specific. As Christensen (2020) describes that latter as the ‘historically dominant, non‐specific friendship model, which holds that a supportive relational bond—alone—promotes positive developmental change [sic] to mentoring’ (p. 959). In contrast, structured, or targeted, mentoring programmes include components to develop specific skills and/or assist with attainment of particular goals (e.g., employment, college acceptance). The relative effectiveness of structured versus unstructured approaches is a key policy issue.

Our review will include only studies on adult mentoring interventions for youth who have offended and at‐risk youth aged under 18 years. Adult mentoring interventions typically pair a youth with a caring adult without advanced professional training who is not a family member to promote positive development of the young person in areas such as behaviour, school performance, and emotional well‐being (DuBois & Karcher, 2013). Adult mentoring to prevent anti‐social and criminal behaviour, including violence, generally involves an adult figure who builds a healthy mentoring relationship and uses it to provides support opportunities for positive path in juveniles. All components and categories of mentoring listed above will be investigated within our review.

### 1.2 How the intervention might work

Existing studies point out the lack of well‐developed theories of change in the design of mentoring programmes, as well as a poor description of mentoring programmes. This limits the knowledge base on mentoring (Tolan et al., 2013). A theory of change for interventions provides insights into how change processes may work.

The theoretical grounding for the review comes from asset‐based approach and the strengths perspective. The strengths‐based approach (Rapp et al., 2006; Saleebey, 2002) contrasts sharply with the deficit‐based approach, which focuses on risk assessment and management (Rapp et al., 2006; Saleebey, 2000). The strengths‐based framework is on the premise that each person, group, family, and community has its own set of assets and that each environment has its own set of resources. It concentrates on recognising and enhancing assets to facilitate positive changes.

In the context of youth engaging in anti‐social behaviours it would be beneficial to test the applicability of this approach to the extent possible. However, quantitative studies may not report the intermediate outcomes of interest to test causal pathways, though qualitative data may provide insights into which ones are most likely to be operating. An asset‐based approach, rather than focusing on what children or youth cannot accomplish, focuses on what they can and do. This technique emphasises positive growth, strengths, and resilience (Rose, 2006). However, quantitative studies may not report the intermediate outcomes of interest to test causal pathways, though qualitative data may provide insights into which ones are most likely to be operating.

Following Rhodes (2005), as described in DuBois et al. (2011) adult‐youth mentoring programmes are believed to work through three channels: (1) A healthy and meaningful relationship is established between the mentor and the mentee. Mentors help mentees build prosocial social behaviours and attachments by providing support and modelling caring behaviour. As a result, mentees' social‐emotional abilities improve. (2) The development of cognitive skills such as information processing and self‐regulation through engaging in discussion with adults. (3) Identity formation, whereby adult mentors act as role models. In addition to these three channels, the following can be understood as building assets: (1) structured programmes may directly contribute to life skills development; (2) mentors act as advocates for children which can help with social and other connections; (3) mentors can provide employment services such as preparing CVs and interview preparation; (4) mentors can assist with connection to services; and (5) there can be a diversionary effect through the time spent with the mentor, and in new interests developed as a result of the mentoring engagement.

According to many studies, the mentor‐mentee bond/relationship is a valuable asset in mentoring (Abrams et al., 2014; Dam et al., 2018; Edwards et al., 2015). Existing reviews on the efficacy of mentoring programmes for at‐risk youth indicate positive outcomes for offending behaviour (DuBois et al., 2002; Rhodes, 2008; Ropasa et al., 2019; Tolan et al., 2014). A theory of change for interventions provides insights into how the change processes unfold. It is intended to explain how activities and immediate and intermediate outcomes are understood to lead to desired changes drawing on causal analysis based on available evidence.

Developing a theory of change for the review will help in understanding if the existing evidence is consistent with the different hypothesised causal mechanisms. In our review, we use the strengths/asset perspective to conceptualise the proposed theory of change as a preliminary attempt to identify assets and protective factors in mentoring relationships that lead to positive outcomes for at risk and offending children and youth.

**Figure 1:**
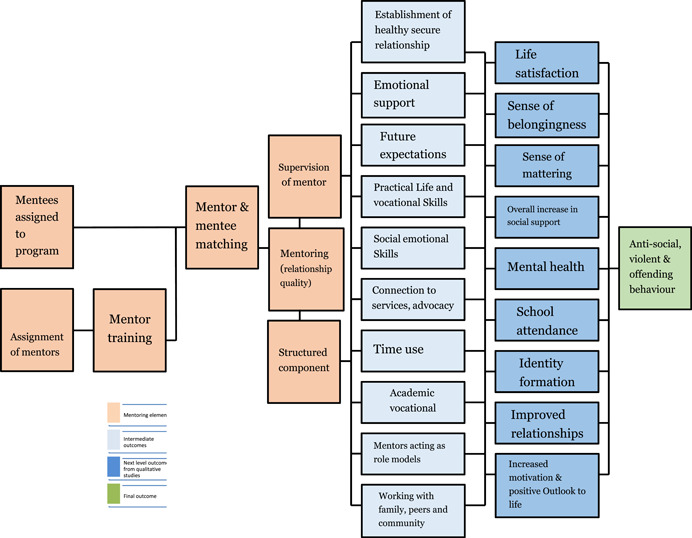
Theory of change for mentoring programmes

There are a range of processes in mentoring that may contribute to the reduction in child anti‐social, violent, and criminal behaviour. In Figure 1, the grey boxes contain the intermediate outcomes. The blue boxes contain the next level of outcomes that eventually lead to behaviour‐related changes. These outcomes will be assessed from the quantitative studies. The proposed theory of change focuses not only on the outputs and outcomes. It also pays attention to the particular elements and processes in mentoring that have been largely overlooked by existing systematic reviews (e.g., DuBois et al., 2011; Raposa et al., 2019).

The orange boxes depict the mentoring programme elements that contribute to reduction in anti‐social, violent and offending behaviour‐related outcomes. That is, mentees assigned to programme, mentors recruited and screened, mentee training, mentor and mentee matching, supervision of mentor and the mentoring intervention that may include a structured component). Theory of change is a participatory process that shows how an intervention is intended to contribute to the desired outcomes by identifying causal links (White, 2009). Hence, an analysis of these elements through the included qualitative studies should facilitate a detailed understanding of the various casual mechanisms.

The theory proposed will be based on papers we will review and additional causal mechanisms which were evident from our scoping of the literature, though not explicitly spelled out as such in those papers.

Such an approach requires a mixed systematic review to look at both effectiveness and implementation issues relating to mentoring and reduction in offending behaviour in children.

The theory of change will be further be refined and also be used to explore and test theories on mentoring (attachment theory, cognitive theory, self‐determination theory, the role of time use, etc.).

### 1.3 Prior reviews

There is only one existing review that assesses the effect of mentoring on offending outcomes (Tolan et al., 2013). This review needs updating.

In addition to that, this review is needed since:

- Youth participation in anti‐social behaviour, violence and offending is a significant cause of concern, with an increase in serious violence reported in recent times.
- Mentoring programmes may address these issues by providing an adult figure who builds a healthy relationship to guide and engage the youth, ultimately providing support opportunities for desired change, reducing the likelihood of criminality, violence, and antisocial behaviour.
- None of the existing reviews are mixed method reviews.
- None of the existing reviews tackle the issue of high rates of attrition which have been observed in mentoring programmes. This attrition has been reported as over 50 per cent in a process evaluation of 80 mentoring interventions carried out by the youth justice board in England and UK between 2001 and 2004 (Robert et al., 2005).
- Raposa et al. (2019) reviewed 70 mentoring programme outcome studies and concluded that mentoring services have a statistically important impact on all youth outcomes considered in the medium/moderate range. The review found that structured mentoring interventions are no more effective than unstructured. However, the review by Christensen et al. (2020) reports that structured mentoring interventions have an overall effect size that is more than double that of non‐specific relational approaches, this conflicting finding will be examined further in this review.
- The funder of this review, the Youth Endowment Fund, is interested in the impact of mentoring on child violence, which has not been assessed in detail in any of the existing reviews Tolan et al.'s (2013) review is dated and reported only aggressions.
- The findings of this review will inform policy makers as to whether mentoring is an effective intervention process to tackle youth offending, and if so, whether this is a cost‐effectiveness approach that should be implemented within future violence reduction strategies. The qualitative evidence presented within our review will also provide extensive insight into the barriers and facilitators to participation and outcomes in mentoring interventions for at‐risk and offending children. This information will be helpful for organisations and professionals involved in mentoring intervention implementation.

## 2 OBJECTIVES

The review will address the following research questions:

- What is the evidence on the effects of adult mentoring programmes in reducing anti‐social, violent and offending behaviour in children aged under 18 years? Are these effects sustained after the end of mentoring?
- Which aspects/features of adult mentoring programmes promote the reduction of anti‐social, violent and criminal behaviour in children aged under 18 years?
- What are the hindering factors/barriers that affect the successful implementation of adult mentoring programmes in children aged under 18 years?
- What are the supporting factors/facilitators that contribute to the successful implementation of adult mentoring programmes in children aged under 18 years?
- What is the evidence on programme costs and incremental cost effectiveness? (The incremental (or marginal cost) is the cost of the providing the intervention over and above the cost of usual services).

### 2.1 Criteria for considering studies for this review

Studies published in English language will be included in the review if they meet the following selection criteria for population, outcomes and study design, as well as being of a mentoring intervention.

### 2.2 Population

Children aged up to and including 17 years who have, or are deemed at risk for, violence offending or anti‐social behaviour. Studies will be included who include youth 17 and below, provided the majority of the intervention and control groups meet the inclusion criteria.

Risk is defined as the presence of personal or environmental traits that raise the likelihood of engaging in criminal or violent behaviour in adolescence or adulthood (Tolan et al., 2013). Children who engage in destructive or violent behaviour, both of which are risk factors for antisocial and criminal behaviour in adolescence, individuals who have suffered traumatic or adverse life experiences, and children from economically disadvantaged families are among them.

Children who have previously offended are especially vulnerable to repeat offences. Mentoring interventions may serve as a correctional intervention for these offending children.

In this review specifically, we will identify ‘at‐risk’ children in a variety of ways: screening before implementing an intervention; formal assessments which sought to identify at‐risk children; referral by social workers; direct recruitment by outreach; geographical targeting; and proxy targeting.

### 2.3 Intervention

- To be included in this review, the mentor must be a ‘formal’ figure, who is recruited for the intervention and not an ‘informal’ mentor figure already present in the life of the at‐risk children (e.g., like a sports coach may be). Mentors must be an adult and not a peer to the at‐risk child.
- Mentoring interventions will be included which are either one‐on‐one or group, and either structured or unstructured.
- Studies of secondary and tertiary adult mentoring interventions are included in this review. These studies are designed specifically for children who are at risk or have already offended. Secondary prevention strategies strive to reduce or eliminate the harm caused by established risk factors, and build on the young person's strengths. They are aimed at those who show early indicators of having poor life trajectories, with the goal of assisting them in achieving a positive life trajectory (Bowen & Neill, 2016).
- Primary mentoring interventions are not a part of this review. Generic mentoring programmes open to all children to navigate a range of challenges such as ‘every child needs a mentor’ in the UK are excluded. We include both studies with mentoring as the sole intervention and studies with mentoring and other interventions. Mentoring and life skills training, mentoring and remedial coaching, and mentoring and sports are examples of multi‐component interventions.
- We will exclude studies with interventions that are entirely focused on the therapeutic component and only have mental health outcomes such as mentoring and cognitive behavioural therapy for depression and mentoring for anxiety disorders.

### 2.4 Outcomes

- To be included in our meta‐analysis, studies must provide a primary outcome to be included. Secondary outcomes will also be extracted from studies included based on their primary outcomes.
- Primary outcomes: violence, anti‐social behaviour and offending (including ‘delinquency’, which is the term commonly used in the US literature).
- Secondary outcomes: Mental health, positive behaviour change, healthy interpersonal relationships.
- Any other studies meeting our inclusion criteria will be assessed to investigate barriers and facilitators: Themes will be extracted from qualitative data. Any quantitative data related to barriers and facilitators, such as participation rates, will also be coded and reported.
- Cost effectiveness data will be extracted from all studies included in this review which report: cost effectiveness, cost per participant, total costs or programme costs.

For full description of outcomes, please see Supporting Information: Appendix A.

### 2.5 Study designs

- This will be a mixed methods review that includes different study designs to address our research questions.
  o To evaluate the effectiveness of adult mentoring interventions (research questions 1 and 2) we will include:
  o Randomised Controlled Trials (RCTs) and non‐experimental/quasi‐experimental designs (designs with a non‐randomly assigned comparison groups).
  o We do not include before versus after studies with no comparison group.
- To understand the success factors and possible barriers to participation in adult mentoring interventions and achievement of outcomes (research questions 3 and 4), we will include:
  o Process evaluations and qualitative studies of interventions: Any evaluation or study of an eligible intervention discussing design and implementation issues.
  o Information on barriers and facilitators were also be extracted from effectiveness studies if reported.
  o To evaluate the cost‐effectiveness of adult mentoring interventions (research question 5), we will include any impact evaluations, process evaluations and cost‐related studies presenting cost data, as well as extracted information from effectiveness studies or process evaluations if available. Cost‐related studies will contain information on cost effectiveness, cost per participant, total costs or programme costs.
- We will include gry literature in this review. This will be achieved by searching conference proceedings within Web of Science and searching for dissertations within Proquest dissertations and Ethos (British Library).
- We will include studies from all years.
- We will include studies from all countries, as long as studies are published in English.
- All settings will be included, other than settings which facilitated interventions focused solely on a therapeutic component and only have mental health outcomes. For example, interventions focusing on cognitive behavioural therapy for depression and mentoring for anxiety disorders.

### 2.6 Search strategy

#### 2.6.1 Electronic searches

The search strategy was developed in consultation with our information specialist (JA). The searches were executed by the information specialist.

##### Databases

Medline, PsycInfo, PsycExtra, Social Policy & Practice, Scopus, Repec, ERIC, Econlit, CASE Engagement database (EEP, UCL), Criminal Justice Abstracts, Criminal Justice Database, Criminology Collection (SAGE) and the US National Criminal Justice Reference Service will be searched.

Supporting Information: Appendix B presents the search strings that will be used for publication databases and search engines, with terms for interventions, regions and methodologies. All searches will restricted to English only, as this is the main language spoken in the review team.

In addition to a traditional, manual database search we will conduct a machine‐learning‐assisted search. The results from the two approaches to database searching will be combined. If key unpublished information is missing from reports of included studies, we will contact lead authors to retrieve this data.

Conference proceedings will be searched for within Web of Science.

Dissertations will be searched for within Proquest dissertations and Ethos (British Library).

#### 2.6.2 Searching other resources

- We will screen the bibliographies of included studies and existing reviews for eligible studies.
- We will also hand‐search the table of contents of the journals listed in Table 1.
- Studies in the YEF Evidence and Gap Map on mentoring will be rescreened for the purposes of possible inclusion in this review.

**Table 1:** List of journals

S. No	Title
1	*Journal of Crime & Justice*
2	*The Journal of Forensic Psychiatry and Psychology*
3	*Victims & Offenders*
4	*Psychology, Crime & Law*
5	*Journal of Offender Rehabilitation*
6	*Deviant Behaviour*
7	*Journal of School Violence*
8	*Journal of Aggression, Maltreatment & Trauma*
9	*Journal of Child & Adolescent Substance Use*
10	*Journal of Evidence‐Based Social Work*
11	*Child & Youth Services*
12	*Journal of Abnormal Psychology*
13	*Psychology of Violence*
14	*Crime & Delinquency*
15	*Journal of Contemporary Crime & Justice*
16	*Youth Justice*
17	*Journal of Research in Crime and Delinquency*
18	*Youth Violence and Juvenile Justice*
19	*Child Maltreatment*
20	*Child and Adolescent Psychiatry and Mental Health*
21	*Journal of the American Academy of Child & Adolescent Psychiatry*
22	*Journal of Youth and Adolescence*
23	*Children and Youth Services Review*
24	*Journal of Applied Social Psychology*
25	*American Journal of Community Psychology*
26	*International Journal of Mentoring and Coaching in Education*
27	*Mentoring & Tutoring: Partnership in Learning*
28	*Journal of Gang Research*
29	*Journal of Social Work Practise*
30	*Victims & Offenders*

In addition, relevant websites listed in Table 2. We will snowball to other websites identified in these searches, systematically documenting each website searched (website, URL, date, any filters or search strings used and studies identified for screening).

**Table 2:** List of websites

S. No	Webpage
1	National Mentoring Resource Center
	https://nationalmentoringresourcecenter.org/
2	The Office of Juvenile Justice and Delinquency Prevention (OJJDP)
	https://ojjdp.ojp.gov/evidence-based-programs
3	Mentoring resource library
	https://www.mentoring.org/resource-library/
4	Youth global justice
	https://www.globalyouthjustice.org/resources/
5	The mentor network
	https://www.thementornetwork.com/program/juvenile-offender-programs/
6	National council for crime prevention (Sweden)
	https://www.bra.se/bra-in-english/home.html
7	UK Justice
	https://www.justice.gov.uk/
8	College of Policing catalogue
	http://www.college.police.uk/
9	European Monitoring Centre for Drugs and Drug Addiction (EMCDDA)
	http://www.emcdda.europa.eu/index.cfm
10	Incredible Years Library
	http://www.incredibleyears.com/research-library/
11	Criminal Justice Research Centre, University of Nottingham
	https://www.nottingham.ac.uk/research/groups/criminal-justice-research-centre/index.aspx
12	Institute of Criminology, University of Cambridge https://www.crim.cam.ac.uk/
13	Scottish Centre for Crime and Justice Research
	https://www.sccjr.ac.uk/
14	Welsh Centre for Crime and Social Justice
	https://wccsj.ac.uk/
15	Prevention collaborative
	https://prevention-collaborative.org/mentors-database/
16	United Nations, library for juvenile justice
	https://www.un.org/development/desa/youth/library-juvenile-justice.html

### 2.7 Data collection and analysis

#### 2.7.1 Selection of studies

Screening of studies for inclusion/exclusion will be undertaken in two stages using EPPI reviewer 4. In the preliminary stage, title and abstract screening will be carried out. The second will encompass full text screening. Both stages of screening will be done by two independent researchers (MN, SS) against predefined inclusion criteria for the review, with a third‐party arbitrator in case of disagreement (HW).

#### 2.7.2 Data extraction and management

For impact and process evaluations and qualitative studies, we will use standardised data extraction forms (Supporting Information: Appendices C and D) to extract descriptive data from all the studies that meet our inclusion criteria. Data extraction from each study will include contextual/geographical information, population, study design and method, intervention types and outcome types, and subcategories. Two researchers (MN, GS) will conduct the data extraction for each study. Both coders will be trained on the tool before starting. Disagreements will be resolved through discussion with a third reviewer consulted as needed (HW). For effectiveness studies, extraction of raw data from evaluations will be conducted by students from Lanzhou University (JL, ZL) and GS. All relevant information will be extracted for all outcomes reported by the primary evaluations, and agreement between the coders will be assessed. Any disputes will be discussed and resolved under the guidance of a fourth reviewer (HW).

### 2.8 Assessment of risk of bias in included studies

The confidence in the study findings of all studies included in the review will be assessed using a critical appraisal tools for primary studies developed by the Campbell Collaboration Secretariat. Please refer to Supporting Information: Appendix E for the tool with coding criteria. The tool for effectiveness studies was first used in the disability EGM published by Campbell (Saran et al., 2020), which used a tool adapted from that of Lund et al. (2010). The qualitative critical appraisal tool is based primarily on the White and Keenan (2018) tool. Coding for critical appraisal will be carried out by two independent reviewers (MN, GS) with disagreements resolved through discussion with a third‐party reviewer (HW). Each researcher was first trained on the critical appraisal tool, and then coded all studies.

The tool contains critical dimensions of the evaluation. Each of these is marked as high, medium, and low. The overall score uses the ‘weakest link in the chain’ principle. Hence, confidence in study findings can only be as high as the lowest rating given to the six critical items in the effectiveness study and nine critical items in the qualitative/process evaluation.

### 2.9 Unit of analysis issues

The primary unit‐of‐analysis for the quantitative data within the studies of interest will usually be the individual that is the specific child within a programme. It is expected that these studies will report data at the programme level, reporting aggregate data for all children in the programme.

Multiple papers or reports based on the same study or data will be treated as a single case for purposes of this review which fits with our proposed approach to mixed methods analysis, described below, in which the unit of analysis is the case, not the paper. That is, a paper report will only be considered as a separate case if the research sample does not include study participants included in any other coded study.

Where there are multiple papers, we will select the most complete reference if all of the relevant information is available in a single source. If the multiple reports each provide different information (e.g., different outcomes or different subgroups), then the data from all these reports will be coded as a single case.

### 2.10 Statistical procedures and conventions

Our study will include some outcomes which are typically reported as dichotomous variables (e.g., offending behaviour), and some which more often reported on the scale (e.g., behavioural measures). To perform the meta‐analysis we will use odds ratios for dichotomous variables and Cohen's *d* for continuous variables.

Odds ratios will be computed via the available information for other effect sizes found in primary studies such as proportions, percentages, raw frequencies, regression coefficients, *χ* ^2^ and marginal distributions, etc. All effect size calculations will be performed using the Campbell online effect size calculator (Wilson, n.d.).

Where an effect, which is predominately reported as a dichotomous outcome, is reported in a paper as a continuous or ordinal measure then the effect size will be calculated as *d*, and then converted to an odds ratio using LOR = *d/*0.5513 (Lipsey & Wilson, 2001).

A random effects model, analogue to the ANOVA approach, will be used to match moderator analyses of a single categorical variable. Metanalytic regression techniques will be used to perform moderator analyses of continuous or multiple moderators, also under a random effects model.

All effect sizes, continuous and dichotomous, will be reported in the common metric of odds ratios converted to a percentage reduction via 2 × 2 table for the purposes of communicating with policy makers and practitioners. For an example of this procedure, see Supporting Information: Appendix F.

### 2.11 Assessment of heterogeneity

Heterogeneity between effect sizes studies will be assessed by reporting the *I* ^2^. Forest plots will be generated for visual representation of pooled effect size on both anti‐social behaviour and offending behaviour. The causes of heterogeneity, if any, will be explored by visual inspection and moderator analysis. Separate forest plots will be presented for important moderators.

### 2.12 Multiple reports of the same outcome

A single study may report the same outcome multiple times for several reasons. We will treat such instances based on the reason for multiple reports as follows:

- Where a study reports multiple effect sizes for the same outcome, we will use the mean of the selected subgroups to ensure that effect sizes were independent, and not given undue weight in our analysis which would bias results.
- For the purposes of moderator analysis, we will code each sub‐group effect size as a unique effect along with details of the sub‐group. A code (full sample or sub‐sample) will be included so that only the full sample estimate is used in the overall meta‐analysis, but the appropriate sub‐sample estimate can be used for the sub‐group analysis.
- Follow up analysis: Where a study has outcome data on follow up, we will code all effects along with the time of the measure. These effect sizes will be used for an analysis of the durability of effects.
- Model specification: Non‐experimental studies may report effect sizes with and without confounders. We will pick the effect size from the preferred model of the study authors (preferred would be the most parsimonious model which allows for confounders). If no preferred model is not stated then we will use the effect size from the most comprehensive model specification.

### 2.13 Intention to treat (ITT) versus treatment of the treated (ToT) outcome measures

High attrition is a problem in many children's programmes. Differential attrition will be reported during the coding stage for all quantitative studies, as it is one of the items in the critical appraisal tool.

Where attrition is high then it matters whether the reported effect size is ITT or ToT. The two should not be combined in a single meta‐analysis. Where a study reports a ToT effect size it will be converted to ITT if the data are available to do so, so that the study can be used in the overall analysis of ITT effects.

### 2.14 Treatment of publication bias

Publication‐selection bias will be assessed for the primary outcomes of anti‐social behaviour, violence and offending behaviour by constructing a funnel plot for each of the two outcomes (Higgins & Green, 2011). The funnel plot will be used for a trim‐and‐fill analysis and the calculation of Egger's test.

### 2.15 Planned moderator analyses

Our a priori planned moderator analyses include the moderators listed in Table 3. Furthermore, we will include as moderators: type of research design (e.g., experiment vs. quasi‐experiment), region of intervention (UK vs. rest of the world), risk of bias and publication type (i.e., published vs. unpublished). Post hoc moderator analyses may be used depending on the analysis of patterns of heterogeneity in the data.

**Table 3:** Potential moderators

Characteristic	Moderator
Country	USA
	Rest of World
Publication type	Published
	Unpublished
Setting of mentoring	Urban Rural
	Urban and rural
Structure of mentoring intervention	Highly structured component
	Moderately structured component Unstructured component
Mentoring vs mentoring plus	Mentoring alone
	Mentoring plus, e.g., academic component
Mentoring component	Mentoring only
	Primary (mentoring is primary component) Secondary (mentoring is secondary component)
Training of mentors	Yes
	No
At risk of offending versus already engaging in offending behaviour	At risk
	Already Offending
Level of risk for offending	Low
	Moderate High
Gender	Male Female
	All sexes
Duration	Length of the mentoring intervention (continuous)
Time of effect analysis	Time taken from the end of the intervention to measurement of the effect
Sample size	All ranges
Intensity	Frequency of meeting
	Time spent per meeting
Age of mentee	All ranges
Age of mentor	All ranges
Ethnicity	All or predominately minority ethnic group (80%+) Partially minority ethnic group (1‐79%)
	No or minority of minority ethnic group (0%)
Nature of intervention	One‐on‐one Group
	Combination of one‐on‐one and group
Research design	Experimental
	Non‐experimental
Mentor mentee matching	Systematic matching
	Random allocation
Type of mentors	Volunteers Paid mentors Teachers
	Probation Officers
Setting for mentoring interventions	School Community House
	other
Key processes in mentoring	Relationship Modelling Emotional support Social support Skills training Guidance
	Advocacy
Termination of mentoring	Majority planned, informed and reported
	Majority unplanned and poorly reported
Study quality	High
	Medium Low
Intention‐to‐Treat (ITT)/	ITT
Treatment on the Treated (ToT)	ToT
Comparison condition	Passive control
	Parole
	Custody
	Alternative mentoring programme
	Alternative treatment

### 2.16 Mixed method analysis (treatment of qualitative research)

Carvalho and White (1997) identify various ways in which qualitative data may be used in an analysis of quantitative data. These ways are similar to those identified in the Cochrane Handbook which states that ‘qualitative evidence synthesis' (commonly referred to as QES) can add value by providing decision makers with additional evidence to improve understanding of intervention complexity, contextual variations, implementation, and stakeholder preferences and experiences’ (Noyes et al., 2019).

This review adopts that approach—that is combining qualitative data with a quantitative meta‐analysis—within the framework of a theory‐based systematic review, TBSR (White, 2018). The TBSR approach—which has similarities with the framework synthesis approach (Booth & Caroll, 2015; Carroll et al., 2013)—takes the intervention as the unit of analysis, not the individual study. Different studies may contribute findings at different stages of the causal chain. For example, process evaluations shed more light on implementation issues than do most effectiveness studies, such as the failure of a quality mentoring relationship to be established and why that was so, which can help explain both the size of, and variations in, effect sizes.

Specifically, qualitative data can be:

- *Integrated* with quantitative data to elaborate the causal chain, that is the different causal mechanisms within the theory of change. For example, there may be a large gap between ITT and ToT effect sizes on account of high attrition as mentors or mentees fail to show up in the first place or drop out. Qualitative data are usually best placed to understand barriers and facilitators to participation.
- Used to *confirm*, *enrich* and *illustrate* the findings of the quantitative analysis. For example, mentoring have can direct and indirect deterrent effects that may lead to reduction in criminal behaviour, aggression and violence. Quotes from young people or their parents supporting these causal mechanisms add colour to the report, strengthening confidence in the effect as one that does operate through the posited causal mechanism.
- Used to *explain* study findings. The TBSR approach uses the funnel of attrition to recognise the fact that effect sizes get smaller moving along the causal chain from outputs to immediate/short‐term and intermediate outcomes to final outcomes.
- The relevant factors in mentoring may include poor relationship with the mentor for various reasons, weak links in the causal chain (e.g., qualitative studies highlight that young offender may not lack self‐esteem, so the causal mechanism through higher self‐esteem through mentoring won't operate), sudden termination of mentoring programmes participation, and that mentoring may actually provide a channel for anti‐social behaviour and aggression.
- The previous point contains examples where qualitative data may contradict or *refute* the intended causal mechanisms, possibly leading to a counter‐theory (Carvalho & White, 2004), e.g., that services for at‐risk children may have iatrogenic effects by bringing them into contact with other anti‐social youth.
- *Merged* with findings from quantitative analysis into a single set of implications for policy and practice.

The TBSR framework is shown in Table 4. Quantitative data are indicated as Qt and qualitative as Ql. Quantitative data refers to both effect sizes and factual quantitative data such as participation rates. As shown in the table, we will test the consistency of the data with various theories identified in the theory of change.

**Table 4:** Stages of the causal chain with data to be examined at each stage

Stage in causal chain	Data
Awareness of the programme amongst relevant service providers and target group	Know of programme, aware of eligibility criteria, purpose and how to access (Qt/Ql)
Enter the programme Stay with programme for whole duration	Attrition (Qt)
	Reasons do not participate or remain in programme (Ql)
Activities undertaken	Descriptive material (Ql)
Informal mentoring role	Mentoring relationship (Ql)
Diversion	Time use (Qt and Ql)
Connection to services	Channels for service connection (Ql)
Behavioural impact	Pro‐social behaviour. Self‐worth. Future outlook. (Qt supported by Ql).
Anti‐social, violent and criminal behaviour	Anti‐social behaviour, aggression, and offending behaviour. Police contacts. (Qt)

Table 4 shows the TBSR framework which is used for both horizontal and vertical synthesis (White, 2018). In Table 2 an abbreviated version of the row headings from Table 1 are pivoted to become column headings. The data in Table 3 are subject to vertical, horizontal, and total synthesis.

Vertical synthesis involves summarising the evidence across all cases, which is the way systematic reviews are usually performed, especially for quantitative analysis of effects. In the case of qualitative data, vertical synthesis is a thematic analysis, in which common themes are identified across studies.

Horizontal synthesis summarises across a case—which may be done in narrative reviews, but with the difference here that the data for an intervention may come from more than one study. The overall synthesis combines, both, though may well contain separate overall synthesis by sub‐group. The overall synthesis approach, drawing on both horizontal and vertical synthesis, ‘tells the story’ of if the intervention works, for whom, under what circumstances and why.

We will use thematic organise the overall synthesis. We will first identify board themes extracted and then reviewed and refine to capture specific barriers and facilitators relating to the objectives of our review (Table 5).

**Table 5:** Theory based systematic framework

Participation	Activities	Mentoring	Services	Behavioural	Offending Behaviour
Case 1					Horizontal
					synthesis
Case 2					
‐‐‐					
Case n					
Vertical					Overall
synthesis					synthesis

### 2.17 Cost analysis

For the cost analysis in the review, we will extract data relating to costs from impact evaluations, process evaluations and cost‐related studies (cost effectiveness, cost‐analysis, and studies that report cost estimates). The data may be an ingredients approach to listing intervention components and their cost, cost effectiveness which includes an estimate of averted cases of offending, or a cost–benefit analysis which sets costs against the financial savings from averted offending or later criminal activity.

The characteristics of these studies will be summarised narratively and in tables. To ensure comparability of cost estimates across studies the costs drawn from studies will be converted to British Pounds (GBP) and then to 2021 prices.

### ROLES AND RESPONSIBILITIES

- Monisha Lakshminarayanan: Monisha is the project lead, responsible for project management, qualitative analysis, report writing, searching, screening, and coding.
- Guy Skinner: Guy will be the second screener and coder and will carry out meta‐analysis under the guidance of HW.
- Patrick Tolan: Patrick Tolan is a Professor at the University of Virginia He will provide support in the overall framework and content of the review.
- David Du Bois: David is a professor at the University of Illinois, Chicago. He will provide technical support with structural equation modelling for the Campbell version of this review and intellectual direction for the review.
- Howard White: Howard is an inter‐disciplinary researcher who has written on the use of mixed methods in a range of subject areas, including systematic reviews (White, 2018). He will provide overall intellectual direction for the review, and provide technical support.

## POTENTIAL CONFLICTS OF INTEREST

Howard White was CEO of the Campbell Collaboration under 31/1/22. He had no role in the editorial decisions regarding this review. Patrick Tolan is lead author of a previous Campbell review on mentoring programmes. There are no other conflicts of interest.

## PRELIMINARY TIMEFRAME

Note, if the protocol or review is not submitted within 6 and 18 months of title registration, respectively, the review area is opened up for other authors.

- Date you plan to submit a draft protocol: June 2021
- Date you plan to submit a draft review: December 2021

## Supporting information

Supporting information.
