# Supplementary material for: PROTOCOL: The effectiveness, implementation and cost effectiveness of mentoring programmes in reducing anti‐social, violent and offending behaviour in children aged 17 years and below: A mixed method systematic review
Source: Campbell Syst Rev. 2022 Nov 12;18(4):e1286. doi: 10.1002/cl2.1286 (PMC9653215; doi:10.1002/cl2.1286)
Supplement: Supplementary file 1 — Supporting information. [file CL2-18-e1286-s001.docx]

**Appendix A**

**Description of outcome categories**

| **Primary and Secondary outcome categories** | **Examples** |
| --- | --- |
| Violent offending | Measured through official records or self-reports: arrests for violent acts; assault; homicide; violent crimes; knife crime. |
| Crime | Measured through official records or self-reports: arrests; delinquency; court contacts; criminal contacts; convictions; non-violent offences; drug arrests; property related crime; misconduct |
|  |  |
|  |  |
|  |  |
| Gang involvement | Self-reported current gang member; ever a gang member |
|  |  |
| Recidivism | Official records reporting recidivism; time to first recidivism; probation officer contacts; likelihood of any reoffending |
|  |  |
|  |  |
| Externalising | Conduct problems (e.g. DAS);CBCL externalising; PSDQ total difficulties; ADHD (e.g. CBCL); ODD (e.g. CADBI); Self-reported or practitioner reported antisocial behaviour, aggression, anger, emotional symptoms |
| Internalizing | CBCL internalizing; PSDQ total difficulties; self-reported, parent reported, or practitioner reported self-esteem, self-concept, self-worth, self-control, self-adequacy, self-regulation, immaturity, coping |
| Attitudes and beliefs | Empathy (e.g. EM scale); self-reported, parent reported, or practitioner reported future orientation, social competencies, acceptance, compliance, attitudes towards older people, hope, attitude to cooperating in crime, attitude to dropping out of school, attitude to having a baby while a teenager, perceptions of classmates’ acceptance |
| Social and emotional outcomes | Thriving (e.g. CIT-CHILD); prosocial skills (e.g. PBQ); developmental assets (e.g. DAF); conscientiousness (e.g. PPTQ-C); social-emotional competence (e.g. EQI:YV) |
| Behavioural outcomes | Prosocial behaviour (e.g.PBS); socialization, responsibility, cooperation, assertiveness (e.g. The HOME Inventory); |
| Substance misuse | Self-reported, parent reported, or practitioner reported alcohol volume; frequency of alcohol use; frequency of drug use; substance abuse; alcohol use; drug use; involved in drug activities |
| Academic and school related outcomes: attendance, attainment, aspirations/attitudes and behaviour | GPA.School reported grades, reading, writing, school delinquency, trouble in school, attendance, discipline referrals, absenteeism, skipping school; self-reported value of school; school delinquency; trouble in school; attendance; discipline referrals; absenteeism; skipping school |
| Familial outcomes | Self-reported, parent reported, or practitioner reported family and living arrangements; special adult; connectedness to family; home support; quality of parental relationship; social support from family, parent, mother, father |
| Peer outcomes | Self-reported, parent reported, or practitioner reported affiliation with delinquent peers; peer refusal skills; connectedness to peers |
| Mental health outcomes | Using standardized measures: depression (e.g. PHQ-9); anxiety (e.g. ASQ); psychotic conditions (e.g. SCL-90-R). Self-reported or medical records indicating mental health treatment. Self-reported well-being |
| Service use, attendance and engagement | Self-reported, parent reported, or practitioner reported community Service; advocacy activities, contracting activities; recreational activities |
| **Barriers and Facilitators** | **Examples** |
| Barriers to participation | Mentor and mentee hesitancies, recruitment processes of mentors and mentees and issues of trust and confidentiality. |
| Facilitators to participation | Mentor characteristics/qualities, healthy mentoring relationship and well-matched mentors and mentees |
| Barriers to outcomes | Communication and coordination issues, poor leadership and funding issues. |
| Facilitators to outcomes | Long term mentoring, successful partnerships and formal termination of mentoring. |
| **Cost Effectiveness** | **Examples** |
| Cost effectiveness | This includes cost-benefit analysis which sets costs against the financial savings from averted offending or later criminal activity. |
| Cost per participant | Information relating to the cost per participant to partake in their mentoring interventions compared with standard service or no intervention. This information will be reported as a daily cost, or a cost for the whole duration of a respective intervention. |
| Total costs | These will be reported in terms of the budgets received, the total costs to deliver a program or the estimated cost to service a population of interest. |
| Program costs | These studies referred to salary costs, costs to offer services, stipends, and incentives to complete interventions. |

n.b.: ASQ: Anxiety Symptoms Questionnaire; CABI: The Child and Adolescent Disruptive Behaviour Inventory; CBCL: Child Behavior Checklist; CIT-CHILD: Comprehensive Inventory of Thriving for Children; DAF: Developmental Assets Framework; DAS: Delinquent Activities Scale; EM: Empathy Scale; EQI:YV: Bar-On Emotional Quotient Inventory Youth; GPA: Grade Point Average; HOME: The Home Observation for Measurement of the Environment; PBS: Prosocial Behaviour Scale; PBQ: Prosocial Behaviour Questionnaire; PHQ-9: Patient Health Questionnaire-9; PPTQ-C: Pictorial Personality Traits Questionnaire for Children; PSDQ: Parental Strengths and Difficulties Questionnaire; SCL-90-R: Symptoms Checklist 90 Revised

**Appendix B**

**Search strategy**

## APA PsycInfo (Ovid) <1806 to January Week 4 2021>Searched 1st February 2021

1 mentor/ or “assistance (social behavior)”/ (11060)

2 mentor*.ti,ab. (17409) 3 or/1-2 (22091)

1. (adolescen* or teen* or youth

or youths or juvenile* or “young people” or “young person*“ or child*).ti,ab. (914878)

1. Early Adolescence/ or exp Predelinquent Youth/ (2438) 6 or/4-5 (914935)
2. behavior change/ or exp behavior disorders/ or exp aggressive behavior/ or exp antisocial behavior/ or exp behavior problems/ or exp criminal behavior/ or exp juvenile delinquency/ or juvenile justice/ or “Adolescent Psychology”/ or exp Adolescent Attitudes/ or exp Adolescent Psychopathology/ or exp Adolescent Psychiatry/ or Adolescent Development/ or treatment outcomes/ or Mental Health Program Evaluation/ or At Risk Populations/ (443278)
3. (delinquen* or anti-social or antisocial or “young offender*“ or “young addict*“ or at-risk or ((disruptive or externali* or criminal or aggressive or violen*) adj2 behavio*)).ti,ab. (127417)

9 or/7-8 (503111)

10 3 and 6 and 9 (**1229**)

## APA PsycExtra (Ovid) <1908 to January 11, 2020>Searched 4th February 2021

1. mentor/ or “assistance (social behavior)”/ (1503)
2. mentor*.ti,ab. (2087) 3 or/1-2 (2504)
3. (adolescen* or teen* or youth or youths or juvenile* or “young people” or “young person*“ or child*).ti,ab. (55913)
4. Early Adolescence/ or exp Predelinquent Youth/ (101) 6 or/4-5 (55914)
5. behavior change/ or exp behavior disorders/ or exp aggressive behavior/ or exp antisocial behavior/ or exp behavior problems/ or exp criminal behavior/ or exp juvenile delinquency/ or juvenile justice/ or “Adolescent Psychology”/ or exp Adolescent Attitudes/ or exp Adolescent Psychopathology/ or exp Adolescent Psychiatry/ or Adolescent Development/ or treatment outcomes/ or Mental Health Program Evaluation/ or At Risk Populations/ (36387)
6. (delinquen* or anti-social or antisocial or “young offender*“ or “young addict*“ or at-risk or ((substance or drug) adj2 (misuse or abuse)) or ((disruptive or externali* or criminal or aggressive or violen*) adj2 behavio*)).ti,ab. (14855)

9 or/7-8 (45551)

10 3 and 6 and 9 (**188**)

## Social Policy and Practice (Ovid) <202010> Searched 4th February 2021

1. mentor*.ti,ab,sh. (1580)
2. (adolescen* or teen* or youth or youths or juvenile* or “young people” or “young person*“ or child*).ti,ab,sh. (109857)
3. (delinquen* or anti-social or antisocial or “young offender*“ or “young addict*“ or at-risk or ((substance or drug) adj2 (misuse or abuse)) or ((disruptive or externali* or criminal or aggressive or violen*) adj2 behavio*)).ti,ab,sh. (19153)

4 1 and 2 and 3 (**188**)

## Econlit (Ovid) <1886 to January 21,2021>Searched 4th February 2021

1 mentor*.ti,ab,hw. (600) 2 J13.cc. (24140)

## [Annotation: Youth Subject Heading]

3 (adolescen* or teen* or youth or youths or juvenile* or “young people” or “young person*“ or child*).ti,ab,hw. (44500)

4 2 or 3 (44500)

5 K42.cc. (13721)

## [Annotation: Illegal Behavior and the Enforcement of Law - Subject heading]

6 (delinquen* or anti-social or antisocial or “young offender*“ or “young addict*“ or at-risk or ((substance or drug) adj2 (misuse or abuse)) or ((disruptive or externali* or criminal or aggressive or violen*) adj2 behavio*)).ti,ab,hw. (6875)

7 5 or 6 (20077)

8 1 and 4 and 7 (**8**)

1. **Ovid MEDLINE(R) and Epub Ahead of Print, In-Process & Other Non- Indexed Citations, Daily and Versions(R) <1946 to February 01, 2021>**

**Searched 4th February 2021**

1. mentors/ or mentoring/ (12664)
2. mentor*.ti,ab,kw. (17237) 3 or/1-2 (23290)
3. (adolescen* or teen* or youth or youths or juvenile* or “young people” or “young person*“ or child*).ti,ab,kw. (1747384)
4. adolescent/ (2065093)

6 or/4-5 (3197595)

1. behavior/ or adolescent behavior/ or underage drinking/ or aggression/ or agonistic behavior/ or bullying/ or problem behavior/ or child behavior/ or criminal behavior/ or dangerous behavior/ or drinking behavior/ or drug-seeking behavior/ or behavior, addictive/ or “marijuana use”/ or marijuana smoking/ or social behavior/ or harassment, non-sexual/ or cyberbullying/ or social conformity/ or juvenile delinquency/ or substance-related

disorders/ or alcoholic intoxication/ or binge drinking/ or cocaine-related disorders/ or inhalant abuse/ or marijuana abuse/ or substance abuse, intravenous/ or substance abuse, oral/ or risk/ or risk-taking/ or risk reduction behavior/ or “risk evaluation and mitigation”/ or “attention deficit and disruptive behavior disorders”/ or conduct disorder/ (473596)

1. (delinquen* or anti-social or antisocial or “young offender*“ or “young addict*“ or at-risk or (risk* adj2 (reduc* or mitigat*)) or ((substance or drug) adj2 (misuse or abuse)) or ((disruptive or externali* or criminal or aggressive or violen* or chang* or disorder*) adj2 behavio*)).ti,ab,kw. (466421)

9 or/7-8 (863401)

10 3 and 6 and 9 (**494**)

## ERIC (Ebsco) – Searched 4th February 2021

S6 S1 AND S2 AND S5 [Database – ERIC]

## 961

S5 S3 OR S4

152,061

S4 DE “Behavior Change” OR DE “Behavior Disorders” OR DE “Addictive Behavior” OR DE “Antisocial Behavior” OR DE “Aggression” OR DE “Bullying” OR DE “Cheating” OR DE “Crime” OR DE “Elder Abuse” OR DE “Hazing” OR DE “Sexual Harassment” OR DE “Vandalism” OR DE “Violence” OR DE “Behavior” OR DE “Behavior Patterns” OR DE “Recidivism” OR DE “Behavior Problems” OR DE “Emotional Disturbances” OR DE “Personality Problems” OR DE “Psychopathology” OR DE “Self Destructive Behavior” OR DE “Addictive Behavior”

84,986

S3 TI ( (delinquen* or anti-social or antisocial or “young offender*“ or “young addict*“ or at-risk or (risk* N2 (reduc* or mitigat*)) or ((substance or drug) N2 (misuse or abuse)) or ((disruptive or externali* or criminal or aggressive or violen* or chang* or disorder*) N2 behavio*))) OR AB ( (delinquen* or anti-social or antisocial or “young offender*“ or “young addict*“ or at-risk or (risk* N2 (reduc* or mitigat*)) or ((substance or drug) N2 (misuse or abuse)) or ((disruptive or externali* or criminal or aggressive or violen* or chang* or disorder*) N2 behavio*))) OR SU ( (delinquen* or anti-social or antisocial or “young offender*“ or “young addict*“ or at-risk or (risk* N2 (reduc* or mitigat*)) or ((substance or drug) N2 (misuse or abuse)) or ((disruptive or externali* or criminal or aggressive or violen* or chang* or disorder*) N2 behavio*)))

105,617

S2 TI ( (adolescen* or teen* or youth or youths or juvenile* or “young people” or “young person*“ or child*)) OR AB ( (adolescen* or teen* or youth or youths or juvenile* or “young people” or “young person*“ or child*)) OR SU ( (adolescen* or teen* or youth or youths or juvenile* or “young people” or “young person*“ or child*)) OR DE “Adolescents”

433,199

S1 TI mentor* OR AB mentor* OR SU mentor* or DE “Mentors” 20,730

## Repec via Ebsco Discovery – Searched 4th Feb 2021

S4 S1 AND S2 AND S3

5,372 **[Limited to Repec – 59]**

S3 TI ( (delinquen* or anti-social or antisocial or “young offender*“ or “young addict*“ or at-risk or (risk* N2 (reduc* or mitigat*)) or ((substance or drug) N2 (misuse or abuse)) or ((disruptive or externali* or criminal or aggressive or violen* or chang* or disorder*) N2 behavio*))) OR AB ( (delinquen* or anti-social or antisocial or “young offender*“ or “young addict*“ or at-risk or (risk* N2 (reduc* or mitigat*)) or ((substance or drug) N2 (misuse or abuse)) or ((disruptive or externali* or criminal or aggressive or violen* or chang* or disorder*) N2 behavio*))) OR SU ( (delinquen* or anti-social or antisocial or “young offender*“ or “young addict*“ or at-risk or (risk* N2 (reduc* or mitigat*)) or ((substance or drug) N2 (misuse or abuse)) or ((disruptive or externali* or criminal or aggressive or violen* or chang* or disorder*) N2 behavio*))

12,591,244

S2 TI ( (adolescen* or teen* or youth or youths or juvenile* or “young people” or “young person*“ or child*)) OR AB ( (adolescen* or teen* or youth or youths or juvenile* or “young people” or “young person*“ or child*)) OR SU ( (adolescen* or teen* or youth or youths or juvenile* or “young people” or “young person*“ or child*))

13,506,659

S1 TI mentor* OR AB mentor* OR SU mentor* 284,227

1. **Web of Science (Social Sciences Citation Index/ Arts & Humanities Index)**

**– Searched 4th February 2021**

# 4 **386**

#3 AND #2 AND #1

Indexes=SSCI, A&HCI Timespan=1970-2021 # 3 241,779

TS=(delinquen* or anti-social or antisocial or “young offender*“ or “young addict*“ or at-risk or (risk* NEAR/2 (reduc* or mitigat*)) or ((substance or drug) NEAR/2 (misuse or abuse)) or ((disruptive or externali* or criminal or aggressive or violen* or chang* or disorder*) NEAR/2 behavio*))

# 2 882,050

TS=(adolescen* or teen* or youth or youths or juvenile* or “young people” or “young person*“ or child*)

# 1 13,832

TS=(mentor*)

## Appendix C

## Screening tool for mentoring

| 1. | Is the paper in English? | No | Exclude |
| --- | --- | --- | --- |
|  |  | Yes | Continue to q2 |
| 2. | Is the paper about an intervention intended to modify the behaviour or attitudes, either directly or indirectly, of children up to the age of 17 who are at risk? | No | Exclude |
|  |  | Yes | Continue to q3a |
| 3. | Is the intervention a secondary or tertiary study on a mentoring intervention which utilizes a formal mentor, who is an adult not a peer or family member? | No | Exclude |
|  |  | Yes | Continue to q3 |
| 3a. | Is the paper a quantitative evaluation reporting measures of eligible outcomes compared to the outcomes (1) in a comparison group (either with or without baseline outcome measures). | No | Continue to q3b |
|  |  | Yes | Continue to q4 |
| 3b. | Is the paper a qualitative process evaluation describing intervention design or implementation, or an analysis of intervention costs? | No Yes | Exclude Include (END) |
| 4. | Do any outcome measuring externalizing, anti- social, conduct disorders or offending behaviour? | No  Yes | Exclude  Include |

**Appendix D**

**Coding tool**

| **Category** | **Subcategory** |
| --- | --- |
| **Publication Status** | - Ongoing - Completed |
| **Region** | - East Asia & Pacific - Europe & Central Asia - Latin America & Caribbean - Middle East & North Africa - South Asia - Sub Saharan Africa - America - Not mentioned |
| **Country** |  |
| **Countries by income** | - Lower- Middle Income Countries - Low- Income Countries - Upper- Middle Income Countries |
| **Settings** | - Rural - Urban - Rural and Urban (Both) - Not Clear |
| **Name of the project/ intervention** |  |
| **Funding agency** |  |
| **Duration of Intervention** | - Less than 6 months - 6 months-1 year - 1-2 years - 2-3 years - More than 3 years - Unclear or not mentioned |
| **Frequency of meetings** | - More than once a week - Once a week - 2-3 times a month - Once a month - Less than one a month - Not clear or not mentioned |
| **Length of meetings** | - Less than one hour - Approximately one hour - 1-2 hours - Over 2 hours - Not clear or not mentioned |
| **Structured element (the extent of direction on conduct of mentoring)*** | - Highly structured - Moderately structured - Lightly structured - Unstructured - Not reported |
| **Unit of delivery** | - Individual-One to one - Group - Combined group and individual |
| **Ages** | - under 9 - 10-14 - 15-17 |
| **Gender** | - Male - Female - Non-Binary - **All sexes** - Not reported |
| **BAME** | - Mainly/exclusively (80%) - Partly - None - Not clear |
| **Risk of offending** | - Low - Medium - High - Not reported |
| **Study Design**** | - Experimental design - Non- experimental design - Process evaluation or qualitative intervention study - Cost analysis |
| **Sample Size** | - Less than 100 - 100-300 size - More than 300 - Not mentioned |
| **Recruitment/Referral mechanisms** | - Service referral - Geographical targeting - School-based - Peer referral - Outreach - Other (state) |
| **Key Professionals involved** | - Volunteers - Paid mentors - Counsellors/ therapists - Teachers - Social workers/case managers - Probation officers - law enforcement authorities - Prison officers |
| **Activities carried out** | - Recruitment of volunteers/ staff - Training of prospective mentors - Systematic matching/pairing of mentors & mentees - Building a supportive & nondirective relationship - Engaging in open & informal conversations - Goal setting - Social & emotional skills building - Spending quality time together & engaging in fun activities - Facilitation of identity development - Family level interventions - School level interventions - Community level interventions - Legal interventions (working with the court, probation officer, prison authorities etc) - Advocacy - Networking (connection to services e.g. employment or legal services) |
| **Setting for mentoring** | - Community - Home - School - Youth centre - Project office - Other |
| **Costs involved** | - Training - Infrastructure - Salaries - Monitoring & supervision - Other - Not mentioned |
| **Components** | - Mentoring only - Mentoring primary component - Mentoring secondary component - Not clear |
| **Intervention sub-category** | **Intervention sub-category (for multi-component approaches)**   - Educational and vocational interventions - Social and emotional interventions - Mental health & therapeutic interventions - Alcohol and drug related interventions - Sports and recreation - Practical life skills - Academic support/Remedial coaching - Others (specify) |
| **Offending related outcomes** | - Violence - Crime/ anti-social activities - Gang membership - Recidivism |
| **Child-centred** | - Attitudes and belief (self-concept, esteem, confidence etc) - Mental health and wellbeing, internalizing behavior and self-regulation, externalizing and risk-taking behavior, and Improved Psycho- social functioning & wellbeing - Substance use - Social outcomes & emotional outcomes (improvement in interpersonal relationships, communication, improved adjustment etc) - Cognitive development- Social Cognition and pro social behaviour - Attainment and knowledge. - Service use, attendance and engagement - Gang involvement |
| **Family & Peers** | - Quality of family relationships and family functioning - Improved interpersonal relationship with peers |
| **Barriers and Facilitators to participation** |  |
| **Barriers and Facilitators to Outcomes** |  |
| **Attrition** | - Drop out _ - Stay on _ - Attrition rate= |
| **Causal Processes** |  |
| **Design issues** |  |
| **Implementation issues** |  |
| **What CYP say** |  |
| **Moderators and Confounders** | - Race - Gender - Socio-economic background   Any other (text box) |
| **Long run impact/sustainability** |  |
| **Costs involved (enter in info box)** | - Total cost - Cost per participant - Cost effectiveness |

Notes:

* Highly structured: Manualized programme with activities and approach prescribed for each session; Moderately structured: Recommended activities and approaches for the mentoring programme, but not session-by-session instructions; Lightly structured: Guidelines provided for mentoring but no prescribed activities;Unstructured: training is provided but no specific requirements for conduct of mentoring.

**Appendix E**

**Critical appraisal tool** (Saran et al.,2020)

| **Critical appraisal tool for primary studies: effectiveness** | | | |
| --- | --- | --- | --- |
| *Item* | **Description** | **Key** | **Notes** |
| ***Intervention*** | Is the intervention clearly named and described, including all relevant components | High: full and clear description, so that the main components and how they are delivered are clear  Medium: Partial description  Low: Little or no description |  |
| ***Evaluation questions*** | Are the evaluation questions clearly stated? | High: full and clear description, so that the main components and how they are delivered are clear  Medium: Partial description  Low: Little or no description |  |
| ***Study design*** | Use the study design coding | High: Experimental  Medium: Non-experimental  Low: Before versus after |  |
| ***Outcomes*** | Are the outcomes clearly defined? Where appropriate do they use an existing, validated measurement tool? | High: full and clear definition using validated instruments where available (a researcher wishing to use these outcomes would have sufficient information to do so)  Medium: Partial definition. May use validated instruments but without sufficient references to source.  Low: Little or no definition |  |
| ***Sample size (power calculation)*** | Do the authors report a power calculation as the basis for sample size? | High: Power calculation report and sample size meets necessary sample size  Medium: Power calculation mentioned and sample size meets necessary sample size  Low: No mention of power calculation. |  |
| ***Attrition*** | Reported for endline and longest follow up.  Calculate overall attrition and differential attrition It is often necessary to calculate from table of results. If sample size varies by outcome calculate for highest attrition. | High: Attrition within IES conservative standard  Medium: Attrition within IES liberal standard  Low: Attrition outside IES liberal standard |  |
| ***Overall (including questions for all studies)*** | The overall score uses the weakest link in the chain principle i.e., is the lowest score on any item | High: High on all items  Medium: No lower than medium on any item  Low: At least one low |  |

**Critical Appraisal tool**

**Process Evaluation** (White & Keenan, 2018)

**Questions for process evaluations (apply to implementation sections) [used for any study coded as having implementation evidence]**

|  |  | **High** | **Medium** | **Low** |  | **Low** |
| --- | --- | --- | --- | --- | --- | --- |
| *1* | Is the qualitative methodology described? | Yes |  | No | >> 3 |  |
| *2* | Is the qualitatively methodology appropriate to address the evaluation questions? | Yes | Partially | No |  | Insufficient detail |
| *3* | Is the recruitment or sampling strategy described? | Yes |  | No | >> 5 |  |
| *4* | Is the recruitment or sampling strategy appropriate to address the evaluation questions? | Yes | Partially | No |  | Insufficient detail |
| *5* | Are the researcher’s own position, assumptions and possible biases outlined? | Yes | Partially | No |  |  |
| *6* | Have ethical considerations been sufficiently considered? | Yes | Partially | No |  | Insufficient detail |
| *7* | Is the data analysis approach adequately described? | Yes |  | No | >>9 |  |
| *8* | Is the data analysis sufficiently rigorous? | Yes | Partially | No |  |  |
| *9* | Are the implications or recommendations clearly based in the evidence from the study? | Yes | Partially | No |  |  |
| *10* | Overall (including questions for all studies- The overall score uses the weakest link in the chain principle i.e., is the lowest score on any item | High: High on all items  Medium: No lower than medium on any item  Low: At least one low |  |  |  |  |

**Appendix F**

**Transforming mean effect size to percentage relative change**

This appendix uses the example of substance misuse outcomes to describe how to estimate the relative reduction from the mean odds ratio.

To transform an odds ratio to a relative change, we first assume 200 youth, evenly divided between treatment and comparison groups. That means there are 100 youth in the control group and 100 youth in the treatment group. Assuming, in this example. that 25% of youth in the control group demonstrated substance misuse, the mean effect sizes can be easily transformed to a percentage reduction in the relevant outcome.

If the odds ratio for substance misuse is 1.392, then using the table below and the formula for an OR, we can estimate the value of X. The odds ratio is estimated as: A*D/B*C, where A is the number of youth in the treatment group who do not demonstrate substance misuse, B is the number of youth in the treatment group that do demonstrate substance misuse, C is the number of youth in the control group that do not demonstrate substance misuse, and D is the number of youth in the control group that do demonstrate substance misuse.

|  |  |  |  |
| --- | --- | --- | --- |
|  | No substance misuse | Substance misuse | Total |
| Experimental | 100-x | X | 100 |
| Control | 75 | 25 | 100 |

Therefore, the value of X is 19.32 and is calculated as follows:

$$\frac{(100-x)(25)}{(75)(x)}=1.392$$

We can then estimate the relative reduction in substance misuse is 22.72% and is calculated as follows:

However, the prevalence of substance misuse is likely to vary between different studies and can be influenced greatly by the type of report (e.g., self-report or observational data) or the time frame (e.g., any substance misuse in the past couple of months versus any substance misuse demonstrated ever), etc. If we were to adjust our assumption that 25% of the control group demonstrate substance misuse, the resulting relative reduction in the treatment group is not greatly affected.

For example, if we assume that 10% of the control group demonstrated substance misuse, the 2x2 table would be as follows and the value of X would be 7.36 and the relative reduction is 26.1%.

|  | No substance misuse | Substance misuse | Total |
| --- | --- | --- | --- |
| Experimental | 100-x | x | 100 |
| Control | 90 | 10 | 100 |

The same calculation is performed for all outcomes with baseline rates of 25%, except reoffending at 50% and violent offending at 17%.
